# Intact imitation of emotional facial actions in autism spectrum conditions

**DOI:** 10.1016/j.neuropsychologia.2010.07.012

**Published:** 2010-09

**Authors:** Clare Press, Daniel Richardson, Geoffrey Bird

**Affiliations:** a Wellcome Trust Centre for Neuroimaging, Institute of Neurology, University College London, 12 Queen Square, London WC1N 3BG, United Kingdom; b Cognitive, Perceptual, and Brain Sciences, Division of Psychology and Language Sciences, University College London, 26 Bedford Way, London WC1H 0AP, United Kingdom; c Division of Psychological Sciences, Birkbeck College, University of London, Malet Street, London WC1E 7HX, United Kingdom

**Keywords:** Imitation, Autism spectrum conditions, Mirror system, Mirror neuron

## Abstract

It has been proposed that there is a core impairment in autism spectrum conditions (ASC) to the mirror neuron system (MNS): If observed actions cannot be mapped onto the motor commands required for performance, higher order sociocognitive functions that involve understanding another person's perspective, such as theory of mind, may be impaired. However, evidence of MNS impairment in ASC is mixed. The present study used an ‘automatic imitation’ paradigm to assess MNS functioning in adults with ASC and matched controls, when observing emotional facial actions. Participants performed a pre-specified angry or surprised facial action in response to observed angry or surprised facial actions, and the speed of their action was measured with motion tracking equipment. Both the ASC and control groups demonstrated automatic imitation of the facial actions, such that responding was faster when they acted with the same emotional expression that they had observed. There was no difference between the two groups in the magnitude of the effect. These findings suggest that previous apparent demonstrations of impairments to the MNS in ASC may be driven by a lack of visual attention to the stimuli or motor sequencing impairments, and therefore that there is, in fact, no MNS impairment in ASC. We discuss these findings with reference to the literature on MNS functioning and imitation in ASC, as well as theories of the role of the MNS in sociocognitive functioning in typical development.

## 1 Introduction

The ‘Broken Mirrors Hypothesis’ (e.g. Williams, Whiten, Suddendorf, & Perrett, 2001) proposes that the core impairment in individuals with autism spectrum conditions (ASC) is to the ‘mirror neuron system’ (MNS) which maps sensory and motor representations of action, and is hypothesised to reside in ventral premotor and inferior parietal cortices. Evidence of MNS impairments in ASC has been taken to support the hypothesis that the MNS plays a role in higher order sociocognitive functions that require us to understand another person's perspective, such as action understanding, theory of mind, and empathy (e.g. Gallese & Goldman, 1998; Rizzolatti & Craighero, 2004; Rizzolatti, Fabbri-Destro, & Cattaneo, 2009). It is proposed that an observed action is translated into motor codes that are used for performing that action ourselves. Mental states driving the actions can then be derived on the basis of mental states that drive our own actions. Under this hypothesis, the social deficits characteristic of individuals with ASC (American Psychiatric Association, 1994) result from damage to the MNS.

Despite the appealing simplicity of the Broken Mirrors Hypothesis, evidence supporting the hypothesis is mixed. Many of the experiments investigating the Broken Mirrors Hypothesis have used imitation tasks, given the evidence that imitation relies on the MNS (e.g. Catmur, Walsh, & Heyes, 2009; Heiser, Iacoboni, Maeda, Marcus, & Mazziotta, 2003; Iacoboni et al., 1999). Although children and adults with ASC perform poorly in a variety of imitation tasks (see Williams, Whiten, & Singh, 2004, for a review) it is not clear whether this is due to specific impairments in the MNS, or impairments in other systems. Most of the imitation tasks used in studies of ASC make substantial demands on a multitude of systems because they assess intentional or ‘voluntary’ imitation. Here, the experimenter asks the participant to copy an action that has many temporal and spatial features, and does not specify exactly which features of the action are to be reproduced. For example, many studies instruct participants simply to ‘do this’ (e.g. Rogers, Hepburn, Stackhouse, & Wehner, 2003). Determining the appropriate action dimensions for imitation, and therefore what constitutes successful performance, is accomplished through the interpretation of subtle cues relating to the social context and the experimenter's mental states. The ability to focus on the selected action dimensions, so that performance is not impaired by imitation of task-irrelevant action dimensions, relies on good theory of mind and understanding of communicative cues, as well as intact executive function and attentional control. There is evidence of impairment to all of these functions in ASC (Bird, Catmur, Silani, Frith, & Frith, 2006; Frith & Frith, 2006; Klin, Jones, Schultz, Volkmar, & Cohen, 2002; Russell, 1997; Southgate, Gergely, & Csibra, 2008). Therefore, impairments to a multitude of systems could result in poor imitative performance in voluntary imitation tasks in ASC (see Leighton, Bird, Charman, & Heyes, 2008).

Stronger evidence concerning impairments to the MNS in ASC comes from automatic imitation tasks and neurological measures. In tests of automatic imitation, participants are not asked to imitate modelled movements. Instead, they are required merely to observe actions, either passively or with a simple movement task, while the experimenter measures involuntary muscular responses (passive observation tasks) or involuntary differences in speed to execute pre-specified actions (simple movement tasks). Both tasks therefore provide a measure of the extent to which observing an action is priming its execution, either through activation of the muscles involved in its execution in a passive observation task, or through greater speed to execute an action when it is preceded by observation of the same action, relative to a different action. McIntosh, Reichmann-Decker, Winkielman, and Wilbarger (2006) used electromyography (EMG) to measure muscular activity in the face while adult participants were presented with emotional facial expressions. Compared with typically developing controls (TD), individuals with ASC showed less expression-compatible muscular activation. That is, when TD controls observed a happy face, they exhibited greater activity in muscles involved in smiling, and when they observed an angry face, they exhibited more activity in muscles used when frowning. The ASC group showed no such pattern. Beall, Moody, McIntosh, Hepburn, and Reed (2008) observed a similar effect in 7–12-year-old children, and Stel, van den Heuvel, and Smeets (2008) in adolescents. Neurological measures have also found that when adults and children with ASC observe facial actions, the typical cortical activation in motor circuits, such as the inferior frontal gyrus, is not seen (Dapretto et al., 2006), or is delayed (Nishitani, Avikainen, & Hari, 2004, see also Oberman, Winkielman, & Ramachandran, 2009) relative to TD controls. As well as group differences, negative correlations have been reported between the level of autistic traits identified in reciprocal social interaction in the autism diagnostic observational schedule-G (‘ADOS’, Lord et al., 2000) and cortical activations in motor circuits when observing action (Dapretto et al., 2006; cf. Beall et al., 2008), such that more atypical reciprocal social interaction is associated with less activity in the MNS. Similar impairments in MNS activation have also been reported when those with ASC observe manual actions (Oberman et al., 2005; Theoret et al., 2005; Williams et al., 2006).

However, in contrast with the findings of impairments to the MNS in ASC, several studies have found evidence that such systems are intact. For example, Bird, Leighton, Press, and Heyes (2007) required adult participants to perform a pre-specified manual action (e.g. open their hand) whenever they observed a stimulus hand perform either a hand-opening, or hand-closing action. This generated trials on which the observed stimulus action was compatible with the executed action (hand opening) and trials on which it was incompatible (hand closing). The degree to which observation of action primed its execution (‘automatic imitation’) was calculated by subtracting reaction time (RT) on compatible trials from RT on incompatible trials. This study found that those with ASC displayed levels of automatic imitation of the manual actions that were equivalent to, if not higher than, levels in the TD control participants. In addition, Gowen, Stanley, and Miall (2008) required adult participants to execute sinusoidal vertical or horizontal arm actions, while watching arm actions in the same or opposite dimension. They found that in both the ASC and TD control groups, variance was higher in the dimension perpendicular to an executed action (e.g. vertical), when observing actions in this opposite dimension (horizontal) rather than the same dimension (vertical), suggesting that observing the actions was activating corresponding motor codes in both groups. Neurological measurements have also shown that when participants with ASC observe manual actions, the primary motor cortex is activated in the same way as in control participants (Avikainen, Kulomaki, & Hari, 1999). Even in voluntary imitation tasks, when the setting is carefully controlled, those with ASC have been sometimes shown to imitate manual actions as accurately as TD controls (e.g. Hamilton, Brindley, & Frith, 2007).

The studies which have and have not demonstrated impaired MNS functioning in ASC tend to differ in two respects. First, the studies finding impairments have tended to use facial actions (but see Oberman et al., 2005; Theoret et al., 2005; Williams et al., 2006), while the studies which have found no impairments have used manual actions. Second, the majority of studies which have found impairments use simple action observation tasks, where actions are observed and incidental motor activations are recorded (behaviourally or neurologically). In contrast, the studies which have found no impairments have implemented motor tasks dependent on observed actions, and measured the degree to which observing action primes execution of matching action.

Previous behavioural (Bach, Peatfield, & Tipper, 2007; Bird & Heyes, 2005; Gillmeister, Catmur, Liepelt, Brass, & Heyes, 2008) and neurological (Buccino et al., 2001; Catmur et al., 2008) studies have indicated that the MNS encodes actions in a body part-specific way. For example, Bird and Heyes (2005) found that when participants observed sequences of actions performed with the fingers, they were subsequently faster to perform these sequences with their fingers, but not with their thumbs. Many studies have indicated that those with ASC do not attend to others’ faces as much as TD controls (e.g. Bird et al., 2006; Klin et al., 2002; Klin, Lin, Gorrindo, Ramsay, & Jones, 2009; Osterling & Dawson, 1994; Riby & Hancock, 2009; cf. Bar-Haim, Shulman, Lamy, & Reuveni, 2006; for a review see Boraston & Blakemore, 2007). Therefore, the lower perceptual input for faces may mean that the MNS representations do not develop in the same way as in TD controls, and that those with ASC have body part-specific impairments in representations of facial actions in the MNS.

The task through which the MNS is studied may also be of importance because of the passive nature of the simple action observation tasks. Specifically, if participants are only required to watch actions, it cannot be assumed that both groups attend to the action stimuli equally, when successful task performance does not require attention to the stimuli. If so, then the individuals with ASC would have exhibited less matching motor activation, even if their MNS were intact. Attentional differences are especially likely in these paradigms, given that the passive observation tasks tend to present facial stimuli which, as previously discussed, may be attended to less by those with autism (Bird et al., 2006; Klin et al., 2002, 2009; Osterling & Dawson, 1994; Riby & Hancock, 2009; cf. Bar-Haim et al., 2006). In contrast, if participants are required to make a response to the stimuli then their level of attention to the stimuli is likely to be higher. The studies that have found no MNS impairment in ASC require participants to attend to the action stimuli in order to perform the task. Therefore, it is plausible that previous findings of apparent impaired MNS function in ASC may have been caused by reduced attention to social stimuli in ASC.

The present study investigated whether it is the task or the body part that determines whether MNS impairments are observed in ASC, to gain a better understanding of whether those with ASC have any impairment to the MNS and attempt to resolve the mixed findings reported in this literature. Adult participants with ASC, and age-, gender-, and IQ-matched control participants, were required to perform a facial motor task dependent on observed facial actions. Participants saw the upper or lower half of a face. The face first appeared in a neutral posture and after a period the eyebrows would raise or lower (if viewing the upper half of the face) or the mouth would open or close (if viewing the lower half), forming half of surprised and angry expressions, respectively. Participants were required to execute a pre-specified response (e.g. raise their eyebrows) whenever the face moved. This generated trials where the response was compatible with the observed movement (eyebrows lifting) and trials where the response was incompatible (eyebrows lowering). As in our previous study (Bird et al., 2007), the RT on compatible trials was subtracted from the RT on incompatible trials to obtain a measure of the degree to which the observed action primed its execution (‘automatic imitation’), and therefore, the representation of this action in the MNS.

If those with ASC exhibit impaired automatic imitation of these facial actions, this would suggest that the body part determines whether impairments are observed, and that those with ASC have body part-specific impairments to the MNS. This would provide support for the Broken Mirrors Hypothesis. In contrast, if those with ASC exhibit intact automatic imitation of these facial actions, this would suggest that previous demonstrations of impairment may be actually driven by those with ASC paying less attention to the action stimuli. This outcome, together with previous findings of unimpaired MNS function in ASC, would provide evidence that those with ASC do not have impairments to the MNS, which would be inconsistent with the Broken Mirrors Hypothesis and suggest that the core impairments in ASC lie elsewhere.

## 2 Materials and methods

### 2.1 Participants

Twenty-eight individuals participated in the study; 14 participants with ASC (11 male) and 14 TD control participants (12 male). Groups were matched on gender, age (ASC M: 41.1 years SE: 3.8 years, control M: 38.2 years, SE: 4.1 years), and IQ (ASD M: 114.4 SE: 3.6, control M 117.1 SE: 2.5). Full-scale IQ was measured using the Wechsler Adult Intelligence Scale-3rd UK Edition (Wechsler, 1999). All participants in the ASC group had previously received a diagnosis from an independent clinician according to standard criteria (see Table 1). The ADOS was used in order to characterize the participants. On this measure, seven participants met criteria for autism, six participants met criteria for autism spectrum condition, and one participant failed to meet criteria (see Section 3). All participants had normal or corrected-to-normal vision and were naive with respect to the purpose of the experiment. The experiment was performed with local ethical committee approval and in accordance with the ethical standards laid down in the 1964 Declaration of Helsinki.

### 2.2 Stimuli

**Fig. 1:**
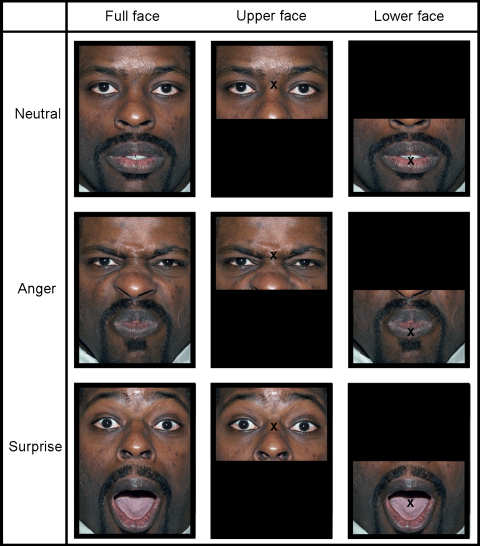
Stimuli were taken from the NimStim set (Tottenham et al., 2009). We used the surprise (mouth open) stimuli for mouth opening and eyebrow raising actions, anger (mouth closed) stimuli for mouth closing and eyebrow lowering actions, and the neutral (mouth open) stimuli. Either top or bottom half of the stimuli were presented, with a fixation cross either between the eyes (upper face stimuli) or centred on the mouth (lower face stimuli).

All stimuli were presented on a computer screen (60 Hz, 400 mm, 96 DPI), in colour on a black background, and viewing was unrestrained at a distance of approximately 600 mm. Stimuli were taken from the NimStim set (Tottenham et al., 2009). There were four tokens (faces 09F, 12F, 30M, and 33M); two male and two female. We used the surprise (mouth open) stimuli for mouth opening and eyebrow raising actions, anger (mouth closed) stimuli for mouth closing and eyebrow lowering actions, and the neutral (mouth open) stimuli. The four tokens were selected from the NimStim set on the basis of large eyebrow and mouth movements for the surprised and angry expressions. The stimuli were trimmed such that it was just facial features that were visible. Either the top or bottom half of the stimuli were presented, with a fixation cross either between the eyes (upper face stimuli) or centred on the mouth (lower face stimuli). The different stimulus types can be seen in Fig. 1, using face 40F from the NimStim set (our chosen stimuli cannot be published in scientific journals). The stimuli subtended approximately 16.8° of visual angle horizontally and 12.2° vertically.

### 2.3 Data recording and analysis

Data were recorded using a Vicon motion tracking system. Markers that were reflective in infrared were placed in the following positions: one on the inner end of each eyebrow, overlaying the corrugator supercilii muscles and therefore detecting eyebrow movements, one on the chin, and therefore detecting mouth movements, and one on the nose as a reference point. The position of each of these sensors was monitored at 360 Hz in *X*, *Y*, and *Z* coordinates, for a 2000 ms period from stimulus movement onset.

The motion tracking data were low pass filtered at 10 Hz. To define a baseline for mouth movements, the mean and standard deviation of the separation between nose and chin sensors was registered for 100 ms when the participant was not moving at the beginning of each trial. To define a baseline for eyebrow movements, the mean of the separations between the nose and each eyebrow sensor was registered. Response onset was defined by the beginning of the first 50 ms window after the imperative stimulus in which all points were more than 10 standard deviations away from the baseline mean, in three-dimensional space. Whether the criterion correctly defined movement onset was verified by sight for every trial performed by each participant by an experimenter who was blind to the trial type.

### 2.4 Procedure

In each block of the simple RT automatic imitation task, participants were required to make the same pre-specified response in every trial, returning after movement to a neutral position with the eyebrows relaxed and the mouth slightly open. They were instructed to make this pre-specified response (to open or close their mouth, or raise or lower their eyebrows) as quickly as possible after the face moved. There was one block for each of the four response action types. Whether eyebrow or mouth actions, and surprise or anger expressions, were executed first, was counterbalanced. Participants were instructed to refrain from moving their face in catch trials, when the face did not move.

**Fig. 2:**
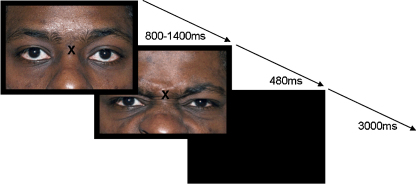
All trials began with presentation of the neutral warning stimulus. In stimulus trials, this was replaced 800, 1000, 1200, or 1400 ms later by onset of the movement stimulus, which was of 480 ms duration. After the imperative stimulus action, the screen went black for 3000 ms before the warning stimulus for the next trial appeared. In catch trials, the warning stimulus remained on the screen for 1980 ms before the 3000 ms inter-trial interval. Participants performed a pre-specified action in each block. In blocks where participants were required to lower their eyebrows, this would be a compatible trial, and in blocks where they were required to raise their eyebrows, this would be an incompatible trial. Order of response actions was counterbalanced across participants.

All trials began with presentation of the neutral warning stimulus. In stimulus trials, this was replaced 800, 1000, 1200, or 1400 ms later by onset of the movement stimulus, which was of 480 ms duration (see Fig. 2). After the imperative stimulus action, the screen went black for 3000 ms before the warning stimulus for the next trial appeared. In catch trials, the warning stimulus remained on the screen for 1980 ms before the 3000 ms inter-trial interval. Each block presented, in random order, 32 stimulus trials and 4 catch trials. There were four stimulus trials of each type, defined by factorial combination of the stimulus action (raising or lowering the eyebrows on upper face stimuli, or opening or closing the mouth on lower face stimuli) and stimulus onset asynchrony (800, 1000, 1200, 1400 ms) variables.

Before testing commenced in each block, participants completed five practice trials (two of each appropriate action stimulus and one catch trial) with the response to be used in that block.

## 3 Results

**Fig. 3:**
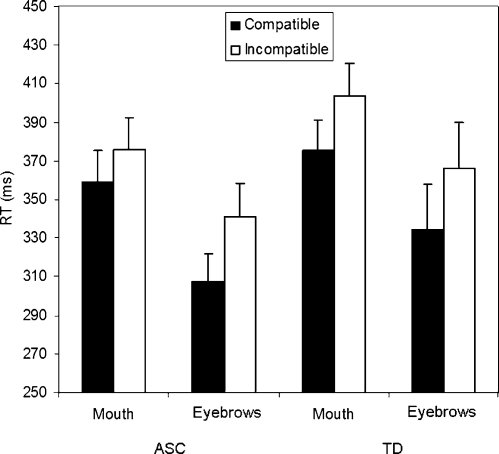
Mean RT (in ms) on compatible and incompatible trials, for mouth and eye actions, in the ASC and TD control groups. Error bars represent the standard error of the mean. The difference in RT between the compatible and incompatible trials is a measure of the degree to which observing an action primes its execution, therefore referred to as the ‘automatic imitation’ effect. Participants with ASC and TD control participants displayed equivalent levels of automatic imitation of facial actions.

Incorrect responses (e.g. mouth opening when closing was required, 0.80%) were excluded from the analysis, as were all RTs smaller than 100 ms and greater than 1000 ms (0.51%), trials on which the participants failed to execute a response (1.34%), trials on which the program did not correctly identify the start of the movement (6.50%), and trials where data failed to capture (1.34%). On each trial, the stimulus movement was either the same as (compatible) or different from (incompatible) the pre-specified response. RT data are shown in Fig. 3.

RT data were analysed using ANOVA with within-subjects factors of compatibility (compatible and incompatible) and body part (mouth and eyes) and a between subjects factor of group (ASC and TD control). This analysis revealed a significant main effect of body part (*F*(1,26) = 8.2, *p* < 0.01), such that eye responses were faster than mouth responses. There was also an effect of compatibility (*F*(1,26) = 43.6, *p* < 0.001) due to faster responses on compatible trials than incompatible trials. There was no group × compatibility interaction (*F*(1,26) = 0.3, *p* = 0.6), with both the ASC (*F*(1,13) = 16.4, *p* = 0.001) and TD control (*F*(1,13) = 29.1, *p* < 0.001) groups demonstrating a compatibility effect. There was also no body part × compatibility interaction (*F*(1,26) = 1.9, *p* = 0.2), no body part × group × compatibility interaction (*F*(1,26) = 0.7, *p* = 0.4), and no main effect of group (*F*(1,26) = 1.3, *p* = 0.3).

Given that one of the ASC participants had only a clinical diagnosis, and failed to meet criterion on the ADOS, the data were re-analysed without this participant. There was still a strong compatibility effect in the ASC group (*F*(1,12) = 17.2, *p* = 0.001), and no sign of a compatibility × group interaction (*F*(1,25) = 0.1, *p* = 0.7). In addition, the participant who failed to meet criterion for ASC on the ADOS displayed one of the lowest automatic imitation effects in the group (4 ms). These data suggest that the equivalent automatic imitation shown by the ASC group was not due to the inclusion of one participant who did not meet ADOS criteria.

## 4 Discussion

The present study found intact automatic imitation of emotional facial actions in individuals with ASC. This finding is consistent with other studies that have found intact automatic imitation of manual actions (e.g. Bird et al., 2007; Gowen et al., 2008), and intact cortical motor activations when observing manual actions (Avikainen et al., 1999) in individuals with ASC. Finding intact automatic imitation of facial actions suggests that there is not a specific MNS deficit for facial actions in ASC. The findings of intact automatic imitation of facial actions in ASC are inconsistent with findings of apparent impairments to the MNS in ASC (e.g. Beall et al., 2008; Dapretto et al., 2006; McIntosh et al., 2006; Nishitani et al., 2004).

We proposed two possible explanations of inconsistencies in the literature concerning possible MNS impairments in ASC. First, the studies finding impairments have tended to use facial actions, while the studies which have found no impairments have used manual actions. This suggests that there may be a face-specific impairment to representations within the MNS in ASC: impaired visual attention to faces (e.g. Boraston & Blakemore, 2007; Klin et al., 2002, 2009; Osterling & Dawson, 1994; Riby & Hancock, 2009; cf. Bar-Haim et al., 2006) may mean that representations of facial actions in the MNS do not develop in the same way as representations of other actions, resulting in a body part-specific impairment of the MNS in ASC. Second, the studies which have found no impairments have implemented motor tasks dependent on observed actions, and measured the degree to which observing action primes execution of matching action. In contrast, a large number of studies which have reported MNS impairments in ASC have simply required participants to observe actions, while involuntary muscular responses, or cortical activations, are recorded. If the participants with ASC did not attend to the actions to the same extent as TD controls in the passive action observation tasks, they would not have exhibited motor activations, even if their MNS were intact. As we found intact automatic imitation of emotional facial actions in ASC in the present study when participants had a motor task to perform which was dependent on observation of the stimuli, it is likely that task demands explain previous mixed findings. If true, this hypothesis suggests that those with ASC do not have impairments to the MNS, but apparent impairments in previous studies have resulted from a lack of visual attention to social stimuli.

Another explanation of previous findings of apparent impairments to the MNS is that those with ASC have problems with motor sequencing. If those with ASC have general problems with sequencing actions one would expect less mirror activity when observing actions. However, under this account, the reduced mirror activity would not be caused by deficits in translating observed actions into corresponding motor commands, but instead by a primary deficit in generating motor sequences. Cattaneo et al. (2007) required children with ASC and TD controls to grasp an object in order to either place it somewhere or to eat it, and recorded the electromyographic response from the muscles involved in opening the mouth during the entire movement. In TD children the mouth muscle was activated several hundred milliseconds before they grasped the food in order to eat it, activated more when they grasped the food, and reaching its peak activation when they opened their mouth. In contrast, the ASC group only exhibited activation in the mouth muscle just before they opened their mouth. Similar patterns were seen during observation such that the TD group exhibited mouth muscle activation when observing early stages of a grasp to eat movement, whereas the ASC group did not. The authors proposed that those with ASC may have problems in chaining motor acts. This hypothesis is consistent with the findings of abnormalities in various motor structures such as the basal ganglia, cerebellum, and parieto-frontal structures in ASC (Brambilla et al., 2003), as well as findings of behavioural motor impairments (Nayate, Bradshaw, & Rinehart, 2005; Rinehart et al., 2006; Teitelbaum, Teitelbaum, Nye, Fryman, & Maurer, 1998). If those with ASC do have problems chaining motor acts, then action observation studies may demonstrate motor system impairments only when the actions require longer sequences than in the present study (notably therefore also requiring dynamic stimuli, e.g. Cattaneo et al., 2007; Oberman et al., 2005), but these impairments would not necessarily be due to a deficit in perception-action matching.

It has been proposed recently that MNS impairments may only apply to some gesture types, such as meaningless gestures, or emotional gestures (e.g. Hamilton, 2009; although see Bird et al., 2007), but not to mirroring of goal-directed actions. The present study indicates for the first time that those with ASC are unimpaired in automatic imitation of emotional facial gestures, therefore demonstrating that they do not have impairments in mirroring emotional actions. Findings of greater impairments in imitating meaningless gestures relative to meaningful gestures in ASC appear widespread (Rogers, Bennetto, McEvoy, & Pennington, 1996; Royeurs, Van Oost, & Bothuyne, 1998; Williams et al., 2004; see also Theoret et al., 2005). However, there are greater sequencing requirements for meaningless actions (e.g. Press & Heyes, 2008; Rumiati & Tessari, 2002; Tessari & Rumiati, 2004), and therefore, this effect may be driven by motor sequencing problems rather than perception-action mapping.

It has been hypothesised that the MNS plays a role in higher-level sociocognitive functioning (e.g. Gallese & Goldman, 1998; Rizzolatti & Craighero, 2004; Rizzolatti et al., 2009). Under this hypothesis, when we observe another person performing an action, we activate the motor commands required to perform that action. This process enables the observer to infer the intentions of the actor, by attributing to the actor the intentions that typically cause the observed action when produced oneself. Evidence that the MNS is impaired in ASC, a population where sociocognitive functioning is known to be impaired, has been regarded as support for this hypothesis. If the MNS is unimpaired in ASC, a significant strand of evidence in support of the role of the MNS in higher-level sociocognitive functioning is undermined (see also Brass, Schmitt, Spengler, & Gergely, 2007). However, it should be noted that it would still be possible that the MNS may be involved in higher sociocognitive functions; the social deficits in ASC may be driven by impairments to alternative mechanisms supporting sociocognitive function.

## 5 Conclusion

The present study has found evidence of intact automatic imitation of emotional facial actions in ASC. This suggests that those with ASC do not have ‘broken mirrors’, and that previously observed impairments in imitation tasks and MNS activation when observing actions may be driven by impaired visual attention to actions or motor sequencing impairments.

## Figures and Tables

**Table 1:** Clinical diagnoses and ADOS-G (subscales and total) scores for the ASC group. Clinical diagnosis refers to the original clinical assessment provided by a psychologist or psychiatrist (A = autism, AS = Asperger's syndrome, and ASD = autism spectrum disorder).

Participant	Clinical diagnosis	ADOS communication	ADOS reciprocal social interaction	ADOS total score
1	AS	2	5	7
2	ASD	4	6	10
3	AS	2	6	8
4	ASD	2	5	7
5	AS	4	6	10
6	AS	3	4	7
7	Atypical A	3	8	11
8	AS	5	10	15
9	AS	1	2	3
10	AS	5	12	17
11	AS	3	6	9
12	AS	4	5	9
13	AS	4	8	12
14	A	4	10	14
